# Malignancies and Biosensors: A Focus on Oral Cancer Detection through Salivary Biomarkers

**DOI:** 10.3390/bios11100396

**Published:** 2021-10-15

**Authors:** Riccardo Goldoni, Alessandra Scolaro, Elisa Boccalari, Carolina Dolci, Antonio Scarano, Francesco Inchingolo, Paolo Ravazzani, Paola Muti, Gianluca Tartaglia

**Affiliations:** 1 Department of Biomedical, Surgical and Dental Sciences, School of Dentistry, University of Milan, 20122 Milano, Italy; riccardo.goldoni@unimi.it (R.G.); alessandra.scolaro@unimi.it (A.S.); elisa.boccalari@unimi.it (E.B.); carolina.dolci@unimi.it (C.D.); Paola.Muti@unimi.it (P.M.); 2 Department of Innovative Technologies in Medicine & Dentistry, University of Chieti-Pescara, 66100 Chieti, Italy; antonio.scarano@unich.it; 3 Department of Interdisciplinary Medicine, University of Medicine Aldo Moro, 70124 Bari, Italy; francesco.inchingolo@uniba.it; 4 National Research Council, Institute of Electronics, Computer and Telecommunication Engineering (CNR IEIIT), 20133 Milano, Italy; Paolo.Ravazzani@ieiit.cnr.it; 5 UOC Maxillo-Facial Surgery and Dentistry, Fondazione IRCCS Ca Granda, Ospedale Maggiore Policlinico, 20100 Milano, Italy

**Keywords:** oral cancer, saliva, biomarkers, biosensors, wearable devices, point of care diagnostics

## Abstract

Oral cancer is among the deadliest types of malignancy due to the late stage at which it is usually diagnosed, leaving the patient with an average five-year survival rate of less than 50%. The booming field of biosensing and point of care diagnostics can, in this regard, play a major role in the early detection of oral cancer. Saliva is gaining interest as an alternative biofluid for non-invasive diagnostics, and many salivary biomarkers of oral cancer have been proposed. While these findings are promising for the application of salivaomics tools in routine practice, studies on larger cohorts are still needed for clinical validation. This review aims to summarize the most recent development in the field of biosensing related to the detection of salivary biomarkers commonly associated with oral cancer. An introduction to oral cancer diagnosis, prognosis and treatment is given to define the clinical problem clearly, then saliva as an alternative biofluid is presented, along with its advantages, disadvantages, and collection procedures. Finally, a brief paragraph on the most promising salivary biomarkers introduces the sensing technologies commonly exploited to detect oral cancer markers in saliva. Hence this review provides a comprehensive overview of both the clinical and technological advantages and challenges associated with oral cancer detection through salivary biomarkers.

## 1. Introduction

**Figure 1:**
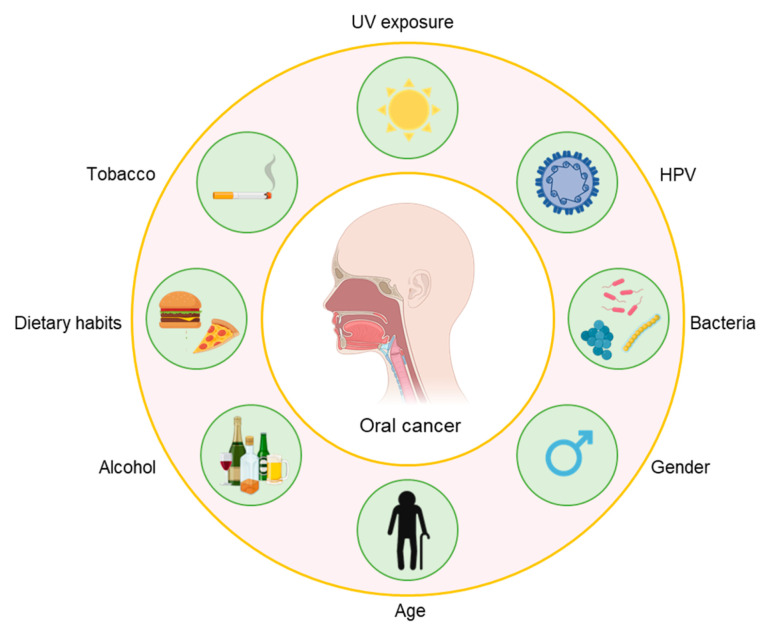
Oral cancer risk factors.

Oral cancer ranks as the 16th most common form of cancer, as 377,713 new cases were estimated worldwide in 2020, counting 177,757 deaths [1]. One third of the total number of oral cancer cases are registered in South-East Asia [2,3,4], mainly due to a rapidly increasing alcohol and tobacco consumption among the population. In fact, several risk factors have been identified for etiopathogenesis of oral cancer and, among these, tobacco and alcohol consumption seem to have a synergistic effect [5,6]. Male sex, old age, high-risk human papillomavirus (HPV), dietary habits, oral bacteria, ultraviolet radiation, as well as betel-quid chewing (commonly used in South-East Asia), have also proved to play a major role in the appearance of the disease [7,8,9] (Figure 1). Moreover, several premalignant lesions, such as erythroplakia, leukoplakia, oral submucous fibrosis and oral lichen planus, have been associated with the onset of the neoplasia [6]. Indeed, a chronic stimulus acting on keratinocytes can lead to hyperplasia, different degrees of dysplasia and then to carcinoma in situ and invasive carcinoma [5].

**Figure 2:**
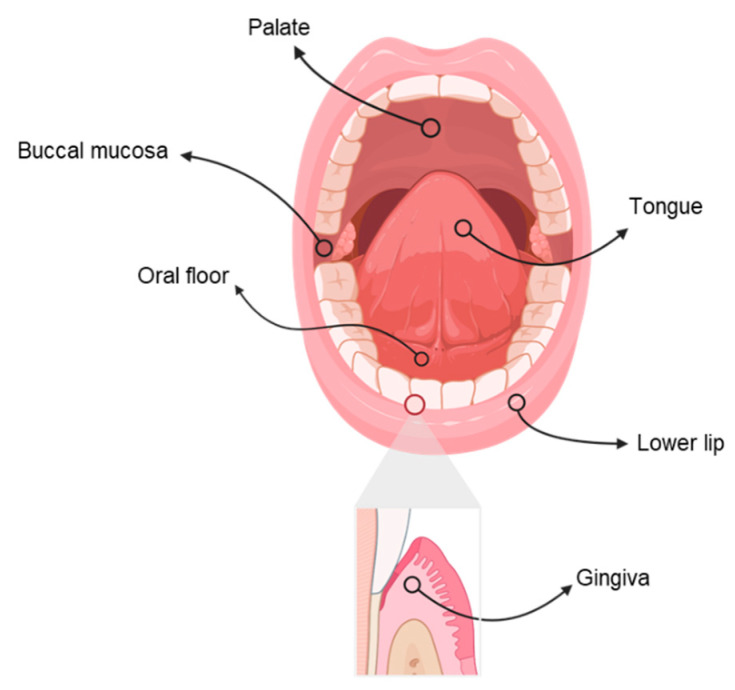
The most common sites of oral cancer.

Oral squamous cell carcinoma (OSCC) accounts for about 90% of these tumors [5,7]. OSCC is an invasive epithelial neoplasia, histologically made by squamous cells that could have several degrees of differentiation [5,6,7]. Figure 2 showcases the sites where oral cancer originates more frequently: these are the tongue, the oral floor, and the lower lip. Other less common locations are the upper and lower gingiva, the palate, and the buccal mucosa [10,11,12,13]. The tumor is able to invade surrounding tissues, such as muscles and bone, and cause distant metastases. In this context, metastases in the cervical lymph nodes are frequent and they can be attributed to the rich lymphatic vascularization of the oral cavity [10].

Oral cancer clinically manifests in several ways and can have variable sizes, up to a few centimeters in advanced cases [11]. Early tumors often appear in the form of erytholeuko-plastic lesions, but they can also primarily manifest as white patches (leukoplakia) or red patches (erythroplakia) and they are usually asymptomatic, thus there is a high risk that they go unnoticed [11,14,15]. OSCC, in its advanced stages, may appear like an ulcerated lesion fixed to underlying tissues, with raised borders and hard on palpation, but it can also be exophytic with a papillary or fungating surface [11,14,15]. At these stages, patients usually suffer from mild or severe pain, depending on the case, that can even spread to the ear [11]. Other signs and symptoms are frequent bleeding, teeth mobility, dysphagia, paresthesia, or even cachexia and severe anemia [11,14]. Advanced forms of OSCC are therefore more easily recognizable with the naked eye, unlike the initial stages of the disease, in which the diagnosis can be uncertain and confirmation by biopsy becomes fundamental [11].

Early diagnosis and treatment of premalignant oral lesions are critically important for reducing mortality [16]. Nowadays, a thorough clinical oral examination and surgical biopsy are considered the gold standards for the diagnosis of pre-malignant lesions and oral cancer [16,17]. More specifically, as suggested by the World Health Organization and the National Institute of Dental and Craniofacial Research, mucosal lesions which persist for two weeks or more after removing possible local irritants should be biopsied [16].

There are two types of biopsies: incisional and excisional and incisional biopsies are the most diffused among the two [15]. Core-needle aspiration, fine-needle aspiration or punch forceps can be used to access more visible lesions, while posteriorly located lesions may require general anesthesia [6]. Although biopsy is the gold standard, it has several disadvantages: the procedure can be extremely stressful, uncomfortable and invasive for the patients; moreover, it involves high costs, a high degree of complexity which require clinician training, and can cause infection or damage to nearby tissues [16,17,18]. On the contrary, cytological examination of a smear is less invasive but also less sensitive and specific than biopsy. Modern cytological procedures, such as brush-biopsy and micro biopsy, could be used to avoid repeated biopsies in the follow-up of precancerous lesions [16].

Non-invasive diagnostic imaging methods, such as CT and MRI, are important to evaluate the extent of tumors and the involvement of lymph nodes, while PET scan could be useful to plan radiation therapy [6,19]. Other diagnostic technologies have been proposed to help distinguish between benign and potentially malignant tumors, detecting cancer in earlier stages, and monitoring the progression of the disease, as well as the response to treatment. Among these, there is an increasing interest in proteomic and genomic techniques, toluidine blue dye, light-base detection techniques, optical imaging systems and detection of biomarkers [16,18,20]. The latter can be carried out in different biological fluids, such as serum, plasma, urine, and saliva, and may be fundamental to screen healthy subjects, allowing an early diagnosis of the disease and monitoring of at-risk patients [21,22,23].

The treatment of choice for OSCC is surgical resection of the tumor [6,7,24,25]. This could be the only treatment modality for stages I and II, while it is usually associated with adjuvant treatment in some conditions, such as larger primary tumors (stages III–IV), involvement of lymph nodes, positive and close margins, as well as perineural and lympho-vascular invasion or extracapsular spread [6,24]. Adjuvant treatment may include radiation therapy and chemotherapy, which can be used both preoperatively and postoperatively in order to help or complete surgical removal [6,7,8]. Other innovative treatments include immunotherapy (anti-CTLA-4 and anti-PD-L1) and the use of an epidermal growth factor receptor (EGFR) antibody, which aims to decrease the amount of EGFR expression, usually associated with a worse prognosis of patients [7,8]. The choice of the best therapeutic strategy depends on the location of the primary tumor, its dimensions, stage and surgical resectability, as well as the patient’s expectations, compliance, lifestyle, and the presence of comorbidities [6,8].

The prognosis of OSCC, depends on several factors, such as patient lifestyle (smoking, alcohol consumption, etc.), presence of comorbidity and the tumor staging [5]. In this context, the TNM system allows definition of the characteristics of the primary tumor, the involvement of lymph nodes and the presence of distant metastases to better clarify the prognosis [6]. Despite advances in treatment options, the five-year survival rate still remains very low, being <50% [7,10]. This can be related to a frequently late diagnosis [5].

The aim of this manuscript is to provide an overview of the state of the art regarding salivary biomarkers for the diagnosis of oral cancer, which are presented along with their common metrics of comparison. The role of saliva in clinical diagnosis is then thoroughly explained, followed by a description of established laboratory techniques for salivary analysis. Lastly, the focus shifts on innovative diagnostic tools, namely biosensors and wearable sensing platforms, which are deemed to be a promising avenue for ultrasensitive and non-invasive early-stage detection of salivary biomarkers related to oral cancer.

## 2. Saliva as Diagnostic and Monitoring Tool

Saliva, which consists for more than 99% of water, contains a variety of electrolytes, including potassium, magnesium, calcium, sodium, bicarbonate and phosphates, as well as biological elements, such as immunoglobulins, mucins, enzymes, proteins and nitrogenous products [26]. The main functions of this biofluid are lubrification, antibacterial activity, buffering action, digestion and tooth protection and clearance [26]. Saliva is produced by two types of salivary glands: the major, which include parotid, submandibular and sublingual glands and are responsible for more than 90% of saliva secretion, and the minor, which are located in the oral mucosa and in the palate [26,27]. The mechanisms concerning salivary glands secretion, however, are still mostly unknown. The amount of saliva volume in individuals not being of a standardized value, the concentration of analytes can change greatly depending on when the sampling/collection procedure is carried out [28].

While blood still remains the gold standard for most examinations, the collection of salivary samples has several advantages: it is non-invasive, simple, stress-free, easily repeatable and it does not require special storage condition or skilled clinicians. Moreover, saliva is stable over time, it does not clot, and it ensures availability of large sample quantities [17,29,30,31,32]. Traditional methods of collecting saliva include burr collection or stimulation methods (e.g., mechanical, gustatory, and olfactory stimuli), which are meant to obtain a larger sample volume. Salivary flow rate is variable, but values greater than 0.1 mL/min are generally considered physiological for unstimulated saliva, while these values increase to 0.2 mL/min for stimulated saliva [26].

Saliva has attracted an increasing interest from the scientific community in the last two decades, thus leading to a large number of recent studies. Many fields, including pharmacotherapy, medicine, and dentistry, have focused on the use of saliva as a diagnostic tool [33]. In this context, the term ‘salivaomics’ aims to include those studies regarding the genome (genomics) [34], as well as RNA (transcriptomics) [34,35], metabolite profiles (metabolomics) [36], proteins (proteomics) [37] and microbial population (micro-biomics) [38]. Among these, the study of salivary genome and epigenome includes the analysis of DNA methylation, which is a stable epigenetic modification that has been associated with systemic conditions, such as chronic kidney disease progression and respiratory allergies [39,40,41]. The detection of specific microRNAs segments is another valuable tool that can be used to diagnose certain systemic conditions. A subset of miRNA sequences, indeed, was repeatedly found to be altered in subjects diagnosed with schizophrenia [42,43]. Another study by Hicks et al. [44] highlighted a specific salivary miRNA profile that was instead reported to be associated with autism spectrum disorder (ASD). More recently Sembler-Moller et al. [45] underlined the overexpression of 14 miRNA segments in Sjogren’s Syndrome patients as compared to healthy controls. miRNA sequences are involved in processes such as cell proliferation, differentiation, apoptosis and gene expression and they can be related to several diseases, including cancer and inflammatory and autoimmune disorders [46,47,48,49]. The analysis of salivary transcriptomic profile concluded that about 117 salivary mRNA species and 3000 mRNA transcripts [50,51] seem to be protected from degradation, unlike blood mRNA species [35]. Moreover, saliva contains specific mRNA and microRNA transcripts which are normally undetectable in blood [52].

Proteomics is a branch of salivaomics that deals with the study of salivary proteins, including those coming from microbial species. More specifically, α-amylases, statherin, P-B peptide, histatins, salivary (“S-type”) cystatins and mucins are the most common markers that can be found in saliva, along with a few proteins, which are mainly secreted by minor glands and gingival crevicular fluid, including α-defensins, b-thymosins and lipocalin [53,54]. In 2003, the creation of a catalog of salivary protein sequences was made possible through the Human Salivary Proteome Project funded by the National Institute of Dental and Craniofacial Research (NIDCR) of the National Institutes of Health [55,56].

Metabolomics and micro-biomics can also benefit from the creation of databases. A database for metabolomics is now freely available and provides information about salivary metabolites and their relative concentrations and associated diseases [57]. Among these, sialic acid, proline, taurine, choline and pipecolic acid are considered useful diagnostic biomarkers for the detection of several diseases [58]. The Human Oral Microbiome Database (HOMD) is, instead, useful to investigate the relationship between microbiota-dependent health and diseases [59]. The oral cavity, possessing the second largest microbiota after the gut, is indeed colonized by a large number of microorganisms [60]. The commensal microorganisms and their host have mutual benefits: the former preventing the adhesion of pathogenic species to the mucosa and modulating the systemic immune defense [61].

**Figure 3:**
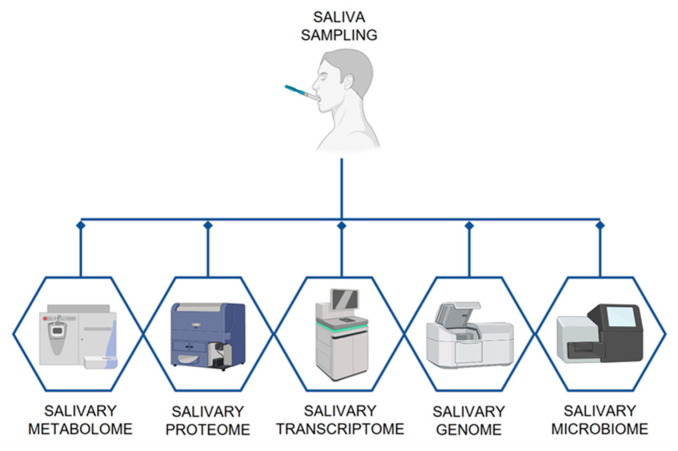
The subfields of salivaomics.

This knowledge advancement in salivary biomarkers has since now largely relied on complex laboratory procedures (Figure 3), such as mass spectrometry, qRT-PCR, microarray chips and deep-sequencing by Next Generation Sequencing (NGS), which are time-consuming and expensive. In this regard, the introduction of point-of-care devices that are able to make diagnosis and rapidly detect salivary biomarkers could be useful to overcome these disadvantages and promote early-stage diagnosis of malignancies.

Obtaining saliva is rapid, simple, and painless, thus making it an extremely convenient fluid to be used for disease screening. However, sample collection must be appropriately optimized to reduce error. Various factors such as assay methods and the standards used affect the results obtained by salivary fluid assessment. The rate of saliva secretion varies among healthy individuals. Since the volume differs among individuals, salivary flow rate and other salivary biomarkers differ from individual to individual.

However, by following proper saliva collection and handling procedures high-quality, reproducible data can be obtained. Before collecting saliva, several factors such as variability of saliva composition, effects of mouth location, effect of flow rate, sample volume and salivary stimulants, salivary analyte and DNA analysis, blood contamination in saliva, saliva collection device and analyte integrity should be carefully considered. 

Proper saliva collection procedures avoid the possibility of contaminating saliva with interfering substances, such as foods with high sugar or acidity, alcohol, caffeine, nicotine, and other factors that could be the source of errors, including prescription/over-the-counter medications taken within the prior 12 h, vigorous physical activity and the presence of oral diseases or injuries.

Various methods for saliva collection are currently available. Depending on the type of sample we want to obtain, we can distinguish between passive and stimulated drool methods. The former is used to collect unstimulated saliva in order to maintain consistency in the type and integrity of sample collected and it is approved for use with almost all analytes. The latter may be used as an alternative collection method for those individuals that have difficulty in carrying out the passive drool technique. As to collection devices, common methods include the draining method, the spitting method, the suction method, and the swab method. Before selecting an appropriate saliva collection method, it is important to consider participant age, analytes of interest, number of samples, sample volumes required, analyte or DNA analysis, self or assisted collection and sample storage. The possibility of sampling error is highest during saliva collection and processing.

Sample contamination can be prevented by wearing gloves and using clean collection materials. The duration and time of sample collection have shown to affect analyte concentration. To overcome sample-to-sample variation, the salivary output of each individual needs to be measured. Specifically, each individual should be classified as secreting a high, medium, or low volume of saliva. The salivary flow rate varies among healthy individuals. To avoid influence of circadian and circannual rhythm, the specimen collection time should be carried out at the same time every day for all individuals, thus minimizing sample variability. Another method for reducing sample variability is to collect samples on multiple days.

Saliva is a highly dynamic biofluid whose composition can vary greatly depending on several confounding factors. Confounding factors are defined as contributions to the composition of saliva coming from the environment or from specific activities (i.e., eating and drinking, smoking, playing sports) carried out by an individual [28]. Other confounding factors relate to low aqueous solubility substances that cannot permeate from the circulatory system into saliva and local enzymatic activities within the oral cavity that can originate secondary byproducts. Standardized analytical methods and collection procedures are the main measures that can be undertaken to minimize confounding factors in salivary analysis.

## 3. Salivary Biomarkers of Oral Cancer

The non-invasive and frequent collection of saliva has enabled the identification of reliable salivary biomarkers for the screening of OSCC. The physio-pathological tissue changes associated with the disease can have genomic, proteomic, and metabolomic expressions. The increasing efforts of the scientific community in the field of salivaomics have produced many studies and more than 100 potential saliva biomarkers for OSCC have already been reported in the literature [18,62,63,64,65].

Cell free DNA (cfDNA), microRNA and circulating tumor cells are among the potential biomarker candidates. They are released into the blood directly from the tumor and most can be found in saliva and thus can be easily sampled. Other potential candidates do not originate from the tumor cells themselves but are instead secreted into the blood as a response of the surrounding tumor microenvironment. These types of biomarker broadly fall into the categories of proteins (mainly secretary) and messenger RNA. 

The classification of biomarkers can be built around several criteria [64]. Diagnostic markers may be present at any stage of cancer development, and they can be specific to tissue, stage, follow-up, relapse, and age. Despite the pressing need to rigorously classify salivary markers for OSCC, as of now a consensus on the topic among the scientific community has still not been reached. Going forward, a promising way to advance the field of salivaomics for OSCC is to identify and validate potential biomarkers while improving the overall ability to detect them by developing technologically advanced biosensors.

### 3.1. Micro RNA (miRNA)

miRNA is small single-stranded non-coding RNA fragments (19–25 base pairs). There are more than 2000 miRNAs encoded in the human genome; they are secreted by cells via exosomes and are able to coordinate cellular and tissue programming by either repression or activation of translation in local or distant cells or by affecting transcription in the cell of origin [66]. miRNA is transferred between cells and thus miRNA fragments can be considered promising potential biomarkers because they convey the initiation and progression of cancer. miRNA signatures have been identified and can be useful to determine tissue-of-origin, stage, progression, prognosis and response to treatment associated with a specific tumor [67].

In a recent systematic review of salivary miRNA and OSCC by Menini et al. [68], a total of 12 studies evaluated salivary miRNA as a diagnostic biomarker, while two studies assessed their relevance for both diagnosis and follow-up. In the two most recent studies included in their review, Yap [69] and He [70] were able to isolate miRNA from saliva and used RT-qPCR and microarray qRT-PCR for genetic analysis. Both papers identified miRNA levels as being significantly increased in OSCC compared to healthy controls. The dysregulation of miR-24-3p was consistently found in these two studies and might signal its usefulness as a target in biosensing applications. 

### 3.2. Cell Free DNA (cfDNA)

CfDNA is a type of DNA that is released into the blood originating from either a healthy cell, tumor cells or cells from the tumor microenvironment. The release occurs due to a number of cellular processes including oncosis, netosis, active secretion, apoptosis, necrosis, and phagocytosis. CfDNA is characterized by base pair length; cfDNA derived from apoptosis in healthy cells is very fragmented, compared to cfDNA derived from necrosis in tumor cells, which tends to consist of longer DNA lengths, typically between 180–200 base pairs, with a half-life of anywhere from 15 min to 13 h.

Classical techniques for sequencing DNA such as Sanger sequencing, qPCR, digital qPCR and massive parallel sequencing (NGS) are not ideal for detection of cfDNA fragment length in saliva. These techniques are either not sensitive enough (Sanger, qPCR) or are too time consuming (NGS).

There have been limited studies evaluating cfDNA as a potential biomarker relative to its detection in saliva of OSCC patients. Shukla et al. [71] evaluated 390 patients using spectrophotometry as the detection method. Within this group there were 90 potentially malignant lesions, 150 OSCCs and 150 post treatment OSCCs, and no significant differences were found between these groups. Wang et al. evaluated 93 head and neck SCC patients using digital PCR finding 76% HPV 16 positivity in saliva. Finally, Schrock et al. [72] evaluated 640 patients with head and neck squamous cell carcinoma (HNSCC) through qPCR and found a 59% positivity rate for methylation markers SOX2 and SPET9. The work done to date suggests that cfDNA is not ready to be used as a full spectrum tumor biomarker for OSCC. 

### 3.3. miRNA vs. cfDNA

In general, miRNA offers several advantages over cfDNA. Among these, as previously mentioned, are that miRNA can identify tissue-of-origin, stage, progression and prognosis and treatment response of a tumor whereas cfDNA essentially gives information on if the tumor is present or not. Secondly, miRNA is far superior to cfDNA in detecting early disease. To generate cfDNA from a tumor it has to reach a size where significant necrosis will be occurring; this of course does not happen in a small lesion to the degree that would produce detectable cfDNA. miRNA signatures can be detected in pre-malignant or dysplastic tissues as well as early malignancies and up to later stages. This early detection allows for early treatment and a higher chance for a positive outcome.

### 3.4. Messenger RNA (mRNA)

Seven cancer-related mRNA biomarkers were consistently validated by qPCR on saliva samples from OSCC patients and controls. These potential salivary RNA biomarkers are transcripts of IL8, IL1B, DUSP1, HA3, OAZ1, S100P, and SAT. The combinations of these biomarkers yielded sensitivity (91%) and specificity (91%) in distinguishing OSCC from the controls.

In recent research by Tang et al. [73] it was shown that long non-coding RNAs (lncRNAs) are expressed across non-tumor, tumor and metastatic tissue samples. Some detected lncRNAs were shown to be aberrantly expressed in cases of oral cancer and metastasis. Moreover, whole saliva contained a detectable amount of some lncRNAs, which appeared to be a potential marker for OSCC.

### 3.5. Protein Biomarkers

Belonging to the large group of protein biomarkers, the proinflammatory and proangiogenic family of cytokines, such as interleukine-6, 8, 1α, 1β have been found to be strong salivary indicators for the diagnosis of OSCC as they usually indicate the carcinogenic transformation from oral precancerous lesions to oral cancer. Katakura et al. [74] found that the expression of these four cytokines was higher in patients with oral cancer than in healthy controls. The difference was significant especially for IL-6, detected through the conventional ELISA technique. Duffy et al. [75] confirmed this observation reporting that serum IL-6 level was also a significant independent predictor of poor survival, as were older age, smoking, cancer site (oral/sinus), higher cancer stage, and comorbidities. 

Krishna et al. [76] reported an abnormal expression of another protein biomarker, telomerase. Telomerase activity was detected positively in 75% of patients with oral squamous cell carcinoma, a notably significant statistical difference when compared a positive rate in 6.67% in healthy controls.

Cytokeratin 19 fragment Cyfra21-1, tissue polypeptide antigen, and cancer antigen 125, were also found to be significantly elevated in saliva samples of OSCC patients that had been analysed with the Immunoblot technique [65]. Moreover, Al Kawas et al. [56] claimed that elevated levels of salivary soluble CD44 were shown in the majority of oral squamous cell carcinoma cases and could distinguish cancer from benign tumors with high specificity. 

Other successfully validated proteins include MRP14, CD59, profilin 1, and catalase. An increased level of MRP14 has been previously reported in tissue cells of oral tongue cancer. CD59 (protectin) is one of the complement restriction factors that are overexpressed in tumor cells, Profilin 1 is a regulator of the microfilament system and is involved in various signaling pathways, Catalase protects the cell against oxidative stress, and altered levels of catalase are fundamentally involved in carcinogenesis and tumor progression. 

Almadori et al. [77] defined Glutathione as an epidemiological marker for chemoprevention and it also indicates a risk of development of OSCC. They showed that salivary glutathione levels may be an index of oxidative stress at the level of the upper airways and of oral cavity and pharynx. M2BP is also used for detection of OSCC, as this biomarker gives a sensitivity of 90% and a specificity of 83% and could also serve as a clinical tool for the non-invasive diagnosis of OSCC.

Further, salivary transferrin is a potential candidate as an early detection biomarker and a prognostic marker for oral cancer, allowing for the development of diagnostic tests. The specificity and sensitivity of salivary transferrin-based ELISA was 95% in overall OSCC patients according to Jou’s research [78]. In addition, the combination of M2BP, MRP14, CD59, profilin, and catalase as candidate biomarkers present sensitivity of 90%, and specificity of 83% in detecting OSCC.

Reddy et al. [79] in a recent research identified the amino acid profile of saliva from patients with oral squamous cell carcinoma using high performance liquid chromatography. The assay levels of amino acids histidine, threonine, valine, isoleucine, methionine, phenylalanine, leucine, lysine, tyrosine, arginine, alanine, glycine, serine, and aspartic acid were significantly higher in both well-differentiated OSCC cases and moderately differentiated OSCC cases, than in healthy controls.

### 3.6. Metabolic Biomarkers

A panel of five salivary metabolites including γ-aminobutyric acid, phenylalanine, valine, n-eicosanoic acid and lactic acid were selected using OPLS-DA model with S-plot [80]. Valine, lactic acid, and phenylalanine in combination yielded satisfactory accuracy (0.89, 0.97), sensitivity (86.5% and 94.6%), specificity (82.4% and 84.4%) and positive predictive value (81.6% and 87.5%) in distinguishing OSCC. The utility of salivary metabolome diagnostics for oral cancer is successfully demonstrated in the study of Wei et al. [80] and these results suggest that the metabolomic approach complements the clinical detection of OSCC.

Lastly, hypoxanthine, guanine, guanosine, trimethylamine N-oxide, spermidine, pipecolate, and methionine have all been reported in elevated concentrations in saliva by Ishikawa et al. [81] and have been used to screen controls from OSCC patients.

Saliva as a diagnostic biofluid has been thoroughly studied for the discovery of biomarkers of OSCC in many patients, and several potential biomarkers in genomic, proteomic, and metabolomics have been detected in the last decade. However, the detection of most of these has been confined to laboratory settings and not expanded into clinics due to issues related to sensitivity and specificity, as well as technical requirements and costs.

## 4. Biosensors and Bioelectronic Platforms for the Detection of Oral Cancer Biomarkers in Saliva

The techniques and methods for cancer diagnosis employed in clinical practice have not substantially changed in the last couple of decades, while many advanced technologies are constantly being developed in laboratory settings. The traditional diagnostic procedure involves two separate stages. At first, an invasives tissue biopsy of the affected region is performed. This is usually followed by medical imaging, including computed tomography (CT), magnetic resonance (MRI) or positron emission tomography (PET) [6,82]. These methods have been validated extensively and are thus widely accepted, but they also suffer from inherent disadvantages such as invasiveness, cumbersome procedures, and high costs [83,84]. Blood tests [85] have opened new avenues for rapid screening of the population and early diagnosis, but they still present a certain degree of invasiveness, especially for fragile individuals.

**Figure 4:**
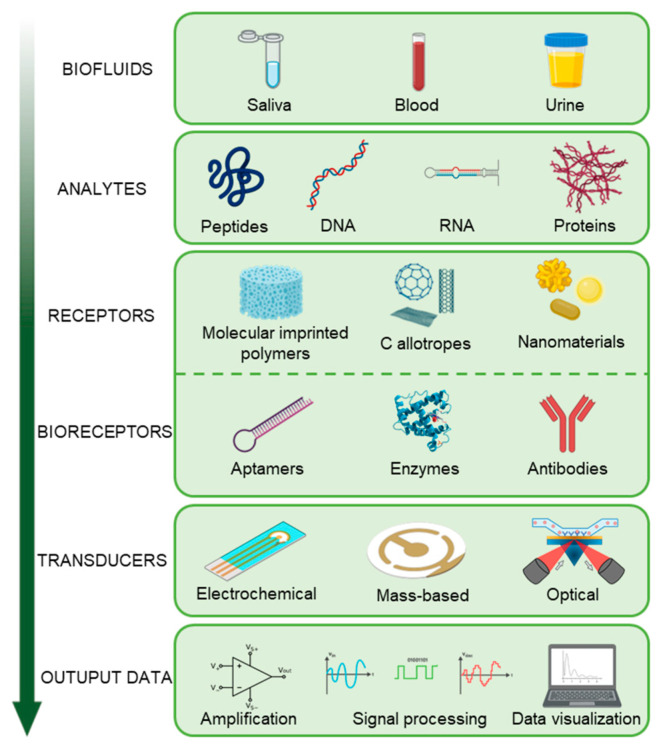
Components of a biosensor and its working principle.

Commercially available ELISA kits provide good sensitivity, but they suffer from an inherently long assay time. Biosensors in this regard offer substantial advantages such as rapid time to response, and easier protocols [86,87]. A biosensor, belonging to the wider category of chemical sensors, is a device that translates a biological event into a measurable signal. The main components of a biosensing system are presented in Figure 4.

A biomarker, or a set of biomarkers, is selected based on the criteria of sensitivity and specificity and then researched in the body fluid of interest. The recognition element serves as the binding site for the selected biomarker, and it can be of natural (bioreceptor) or synthetic (receptor) nature, or a combination of both. The transducer then operates the conversion of the biological signal generated by the binding event into a measurable signal, through a specific detection mechanism (electrochemical, mass-based, optical, etc.) that also depends on the biomarker. The signal obtained is then amplified and undergoes several steps of signal conditioning and processing, either on the device itself (on-board) or after the signal has been transmitted to a remote device.

The possibility to non-invasively analyze body fluids, including serum and saliva, expanded the library of available biofluids for earlier diagnosis and timely treatment of malignancies [88,89]. The field of biomarker discovery has largely relied on serum or plasma as the biofluid of choice [85,90,91]. Saliva is a readily available biofluid that, as a biomarker resource, has been relatively unexplored until about a decade ago. Then D.T. Wong and his research group pioneered the use of saliva as biofluid for oral cancer biomarkers detection [92,93,94,95,96] and since that moment the field has seen a marked increase in interest from the scientific community [92].

The rapid advances in flexible electronics and miniaturized technologies now allow the development of versatile and scalable bioelectronic platforms that could be employed for early cancer screening, from readily available body fluids [97]. The identification of changes in saliva indicating disease progression underlines the utility of saliva as a non-invasive source of informative biomarkers reflecting disease burden and progression. Saliva also possesses drawbacks, including rapid biofouling on the surface of biosensors [98], the effect of interferents that are present in saliva in different concentrations [99], and the presence of a highly dynamic environment orally. Nevertheless, the development of advanced bioelectronic tools have enabled researchers to overcome most of the bottlenecks that are present today in salivary diagnostics.

Table 1 summarizes the most recent efforts in the development of biosensors for early diagnosis of oral cancer. The year of publication has been included to present the reader with the evidence of an increasing interest among the scientific community toward oral cancer detection through biosensors targeting saliva. Quantitative performance parameters such as limit of detection (LOD) and response/incubation time have been reported as a metric of comparison between the studies. In most cases, raw saliva is the only biofluid that has been analyzed to clinically validate the developed biosensor. However, there are studies where the authors decided to evaluate the device performance in both artificial and real saliva, in order to estimate the matrix effect of a real biofluid as opposed to the artificial one [100,101,102,103,104,105,106,107].

Biosensing for salivary diagnostics still being a rather burgeoning research field, it is of great importance also to validate the obtained results against an established gold standard, such as ELISA. Based on the data that have been gathered and reported in Table 1, only half of the studies have been validated against a gold standard. Moreover, most of the studies including a gold standard validation come from the same research groups, highlighting how clinical validation is still a practice that depends on individual laboratory protocols.

**Figure 5:**
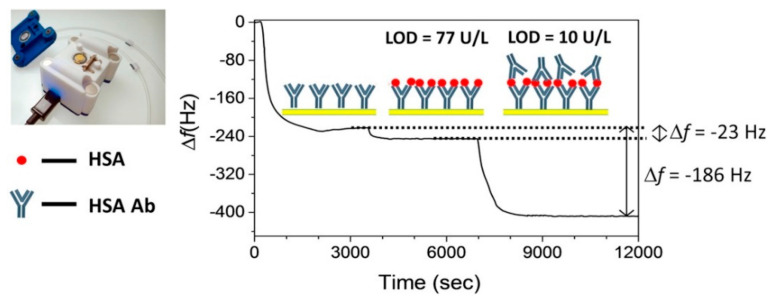
QCM-based biosensor for human salivary α-amylase determination. The immobilization of the various components and targets on the QCM surface is accompanied by characteristic shifts in frequency that are quantified by the readout system. Reprinted with permission from ref. [131]. Copyright 2017 Elsevier.

A variety of detection mechanisms have been employed in biosensing, including electrochemical [111,112,113,115,116,118,119,121,122,125,129,133,135,141], photoelectrochemical [114], optical [100,110,117,124,126,127,140], and mass-based systems [131]. All of the above biosensing methods enable detection with high sensitivity, and enhanced specificity [142]. Mass-based systems, like the one reported in Figure 5, are inherently versatile in terms of the biomarkers they can detect since they are only based on a mass variation upon binding of the target molecule on the surface of a quartz-crystal microbalance (QCM). QCM biosensors exist in different sizes, hence they can fit different applications including miniaturized electronic devices. An inherent drawback of this type of system is the susceptibility to being disturbed by external events, such as vibrations and mechanical stress. As a consequence, QCM biosensors are rarely employed in wearable or portable systems.

Della Ventura et al. [131] realized a robust QCM-based mass sensor to detect the concentration of human salivary α-amylase (HSA) in human saliva and total α-amylase (the HSA and human pancreas amylase) in urine and serum. The group exploited the use of an unconventional technique to functionalize the sensor’s surface, called the photochemical immobilization technique (PIT). By means of PIT functionalization it was possible to have a favorable orientation of the antibodies on the sensor’s surface, enhancing the performance of the device. The detection of HAS in saliva was performed through a simple one-step dilution process. By adding a second step consisting of a ballasting procedure, it was also possible to adapt the system to detect the concentration of total α-amylase in urine and serum, greatly enhancing the sensitivity and lowering the LOD.

### 4.1. Electrochemical Biosensors

Electrochemical biosensors have seen a larger adoption compared to other sensing technologies since they are suitable for the detection of almost any kind of biomarkers, and they can be easily integrated with traditional laboratory benchtop equipment. Moreover, they are prone to be integrated into wearable and portable devices since they can be easily miniaturized [143,144,145,146]. Integration of electrochemical sensors into small sized device has to satisfy stringent requirements of convenience, comfort, simplicity of operation and flexibility, hence the development of reliable wearable and portable point of care (POC) ultrasensitive devices still remains challenging.

**Figure 6:**
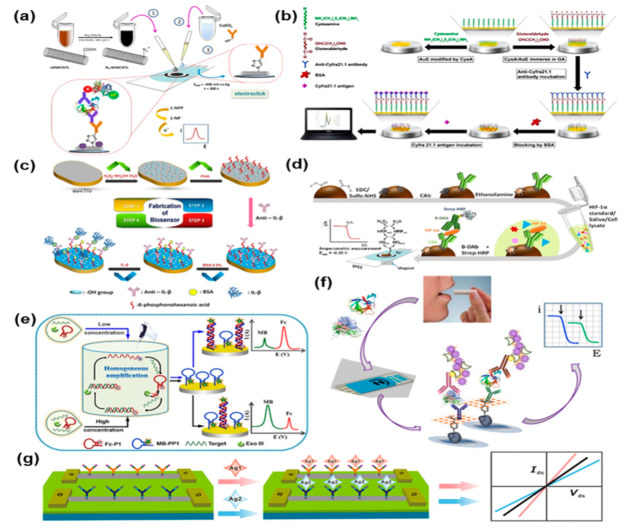
Summary of recently developed electrochemical biosensors for the detection of oral cancer biomarkers in saliva (**a**) Sandwich-type immunosensor for the detection of interleukin 1β (IL-1β) in saliva through DPV. Reprinted with permission from ref. [111]. Copyright 2020 Elsevier. (**b**) Immunosensor for Cyfra21.1 detection based on a cysteamine and glutaraldehyde modified gold electrode. Reprinted with permission from ref. [112]. Copyright 2020 Elsevier. (**c**) Label-free immunosensor based on a modified ITO electrode for EIS detection of oral cancer biomarker in both saliva and serum. Reprinted with permission from ref. [119]. Copyright 2018 Elsevier (**d**) Magnetic beads-based sandwich immunoassay for the ampero-metric detection of HIF-1α. Reprinted with permission from ref. [115]. Copyright 2020 Elsevier. (**e**) Ratio-metric electrochemical DNA biosensor for the detection of ORAOV1 in saliva. Reprinted with permission from ref. [102]. Copyright 2018 Elsevier. (**f**) Multiplexed immunosensor based on dual SPCEs for the simultaneous detection of IL-1β and TNF-α. Reprinted with permission from ref. [130]. Copyright 2017 Elsevier. (**g**) SiNW sensor array for the multiplexed detection of TNF-α and IL-8. Reprinted with permission from ref. [107]. Copyright 2015 The Japan Society for Analytical Chemistry.

Figure 6 provides an overview of recently developed electrochemical standalone sensors for the detection of oral cancer biomarkers. A plethora of different approaches have been used to functionalize the surface of the sensing electrodes, including the use of antibodies [103,104,105,107,109,111,112,116,118,119,120,121,122,124,125,128,129,130,131,132,134,136,137,138,141], magnetic beads [115,135,138,140], and aptamers [101,126,133].

Guerrero et al. [111] recently developed an electrochemical sandwich-type immunosensor (Figure 6a) through an electro-click methodology for the detection of interleukin 1β (IL-1β) in saliva. The group utilized differential pulse voltammetry (DPV) as their preferred detection technique. Disposable screen-printed carbon electrodes (SPCEs) have been modified with azide-modified-multi-wall carbon nanotubes (aMWCNTs). Through the addition of a secondary label for signal amplification the sandwich immunosensor reached a lower LOD of 5.2 pg/mL. substantially superior compared to a commercial ELISA kit. Moreover, the total time-to-results of the developed immunosensor was more than one hour shorter than that of ELISA.

Today more than a hundred potential biomarkers have been linked to oral cancer by different researchers [147]. Among these is a member of the cytokeratin family, a fragment of cytokeratin-19 called Cyfra21.1, that has been extensively researched in saliva by means of biosensors [104,105,109,112,120,124,134,136,137]. Jafari et al. recently [112] built an immunosensor (Figure 6b) by immobilizing anti-Cyfra21.1 on a cysteamine (CysA) and glutaraldehyde (GA) modified gold electrode. The performance of the biosensor was investigated through square wave voltammetry (SWV) and validated with a commercial ELISA test. The group reported a LOD of 2.5 ng/mL and a linear range between 2.5 and 50 ng/mL. The platform provides a low-cost, reliable, and robust method to perform non-invasive detection of salivary Cyfra21.1.

Aydin et al. [119] realized a label-free immunosensor (Figure 6c) based on a modified indium tin oxide (ITO) electrode to detect IL-1β in both saliva and serum. This study pioneered the use of 6-phosphonohexanoic acid (PHA) as a biomolecule immobilization matrix to attach anti-IL-1β for selective binding of the analyte. A variety of electrochemical techniques has been employed to verify the conditions at each fabrication step and PHA proved to be a suitable material for a reliable and stable operation of the biosensor. The electrochemical immunosensor was studied through electrochemical impedance spectroscopy (EIS) and the metrics of stability, repeatability, reproducibility, and specificity have been assessed and compared with the state of the art. The impedimetric immunosensor reached a LOD of 7.5 fg/mL in a 0.025–3 pg/mL concentration range.

Munoz-San Martìn et al. [115] reported for the first time the use of magnetic beads (MBs) in an electrochemical assay (Figure 6d) for the ampero-metric detection of hypoxia-inducible factor-1 alpha (HIF-1α). HIF-1α is involved in tumoral hypoxia and has been identified as a potential biomarker for the prognosis of oral squamous cell carcinoma (HSCC) [148]. The biosensor has been designed with a sandwich configuration to require few incubation steps, thus reducing the overall assay time compared to established laboratory techniques. The group tested the device in buffer solutions against commonly encountered interferents (human IgG, BSA, TNF-α, etc.) and in real human saliva samples. The biosensors exhibited a low LOD of 76 pg/mL in a short assay time of only 105 min. The compact size and ease of operation of the developed immunosensor makes it promising for POCT applications.

Another approach for the detection of salivary biomarkers involves the use of DNA biosensors. Ma et al. [102] realized a ratio-metric electrochemical sensor (Figure 6e) for the detection of Oral Cancer Overexpressed 1 (ORAOV1), by coupling homogeneous Exo III-aided target recycling amplification and one-step triggered dual-signal output. This strategy aims to overcome the usual limitation of conventional electrochemical biosensors based on signal-on/signal-off outputs. The research group was able to develop the device and test it in spiked saliva samples, showing promising results for its clinical validation. They obtained a LOD of 12.8 fM within a linear range of 0.02–0.2 pM and verified the selectivity of the device against similar DNA sequences.

While in most cases the detection of a single biomarker is sufficient to discern between a healthy patient and a sick one, the capability to detect two different biomarkers simultaneously, known as multiplexing, allows for even more precise outcomes. Sanchez-Tirado et al. [130] realized an immunosensor (Figure 6f) based on dual SPCEs for the detection of IL-1β and TNF-α, two oral cancer biomarkers. The complex immuno-platform utilizes carboxy-phenyl-functionalized double-walled carbon nanotubes (HOOC-Phe-DWCNTs) as a substrate to separately immobilize the antibodies for each biomarker. The use of a commercial polymer coating allowed for a better antibody immobilization. The addition of poly-HRP-streptavidin conjugates, used for signal amplification, completed the development of the sandwich-type immunosensor. The group was able to realize a sensitive ampero-metric biosensor with a relatively low-cost and simple process, achieving a LOD of 0.38 for IL-1β and 0.85 for TNF-α, within a linear range of 0.5–100 and 1–200 pg/mL, respectively. The assay time reported is also shorter than commercial ELISA, also considering the detection of two biomarkers at the same time.

Field effect transistors (FET), while less common than two and three-electrode setups in electrochemical sensing, are gaining attention due to their high sensitivity. Among these, silicon nanowire (SiNW) FETs have demonstrated bio affinity toward biomarkers and the possibility of performing a multiplexed detection by selective functionalization of the nanowire with bioreceptors. Zhang et al. [107] realized a SiNW sensor array (Figure 6g) to detect TNF-α and IL-8 simultaneously, taking advantage of their intrinsic opposite charge, allowing for a simple discrimination. Their biosensor has been validated in artificial saliva, reaching a LOD of 100 fg/mL in a short time, providing a promising tool for early detection of OSCC through salivary diagnostics.

### 4.2. Optical Biosensors

**Figure 7:**
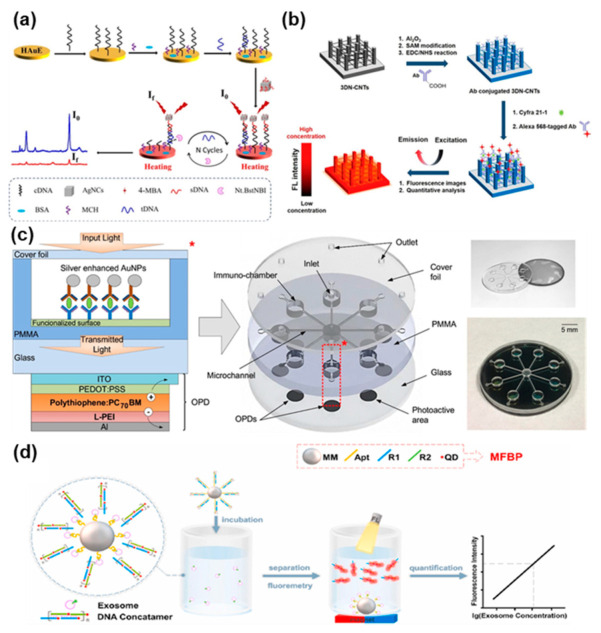
Summary of recently developed optical biosensors for the detection of oral cancer biomarkers in saliva (**a**) SERS-based optical DNA biosensor. Reprinted with permission from ref. [100]. Copyright 2021 Elsevier. (**b**) Fluorescence immunosensor based on 3DN-CNTs for the detection of Cyfra21.1. Reprinted with permission from ref. [124]. Copyright 2018 Elsevier. (**c**) Absorbance-based biosensor with integrated microfluidic channels and antibody-functionalized detection chambers. Reprinted with permission from ref. [127]. Copyright 2017 Elsevier. (**d**) Detection of salivary exosomes through magnetic and fluorescent bio-probes for the non-invasive diagnosis of oral cancer. Reprinted with permission from ref. [110]. Copyright 2021 Elsevier.

Optical systems provide another opportunity to perform salivary analysis with high sensitivity, selectivity, and label-free operation. Many non-invasive optical technologies have been developed in the past, and they can be divided into fluorescence-based biosensors, surface-enhanced Raman spectroscopy (SERS) biosensors, photonic crystal biosensors and surface plasmon resonance (SPR) biosensors [149]. Biosensors based on colorimetry can also be classified as optical sensors and they offer a qualitative and rapid way to perform non-invasive diagnostics on biofluids. Optical devices often include microfluidic channels that ensure the biofluid is delivered in the area where the detector/reader is positioned, for a precise and repeatable analysis [150]. The most representative research articles featuring optical biosensors for salivary diagnostics of oral cancer biomarkers have been reported in Figure 7.

Among the optical methods of detection listed above, SERS is one of the most frequently employed analytical techniques. This is mainly due to its high sensitivity, versatility, ease of operation and multiplexed capability [151]. In this regard, the use of nanostructured materials is often sought to increase the sensitivity of the device. Liu et al. [100] developed a biosensing platform (Figure 7a) based on plasmonic Ag nano-cubes (AgNCs) as a strategy to enhance the SERS signal. The platform combines nicking endonuclease assisted signal amplification (NESA) with heated electrodes for the detection of a DNA sequence related to oral cancer. The Nt.BstNBI/AgNCs/HAuE DNA biosensor showed high sensitivity within a linear range of 10 fM–1 nM and a cleavage time of only 1 h.

Song et al. [124] developed a fluorescence immunosensor (Figure 7b) to detect Cyfra21.1, an oral cancer biomarker. This novel biosensor is based on a Si pillar substrate. A 3D network of antibody-functionalized carbon nanotubes (3DN-CNTs) is then created among these Si pillars and, through the use of a specific tag, fluorescence images are obtained. The research group validated the developed biosensor in real human saliva samples with an established technique, such as electrochemiluminescence (ECL), to prove its clinical applicability, obtaining a good correlation between results.

The combination of microfluidic systems and optical sensing platforms has proved to be a strategic choice to obtain simple and rapid detection of analytes in several biofluids. Dong and Pires [127] developed a microfluidic biosensor (Figure 7c) based on absorbance for the multiplexed detection of three salivary biomarkers commonly associated with oral cancer, IL-8, IL-1β and MMP-8. The biosensor has been designed based on criteria of compactness and miniaturization, in order to present the reader with an attractive tool for POC diagnosis. Organic photodetectors (OPDs), representing the photoactive area, are realized on a glass substrate, and vertically aligned with the microfluidic polymeric channel, featuring chambers with antibody-functionalized gold-silver immunoassays. The group obtained LOD in the pg/mL range within a wide linear range. They also validated the obtained results with commercially available ELISA tests to prove its utility in clinical practice.

Wu et al. [110] focused on the detection of salivary exosomes as a promising source of biomarkers related, among other malignancies, to oral cancer. Specifically, they developed a fluorescent biosensor (Figure 7d) based on magnetic and fluorescent bio-probes (MFBPs) loaded with quantum dots (QDs) for an efficient signal amplification. Specific aptamers were immobilized on the surface of magnetic microspheres (MMs) to selectively bind CD63 proteins on exosomes. The shape changes of aptamers following the binding event released QDs-conjugated tethered DNA concatemers for signal amplification. They reached a lower LOD of 500 particles per microliter of solution and tested the device both in buffer and real human saliva samples, to precisely evaluate the matrix effect. In order to validate their experimental findings, the research group also performed the tests on a NanoFCM analyzer, obtaining a good correlation between the two methods.

### 4.3. Integrated Systems

**Figure 8:**
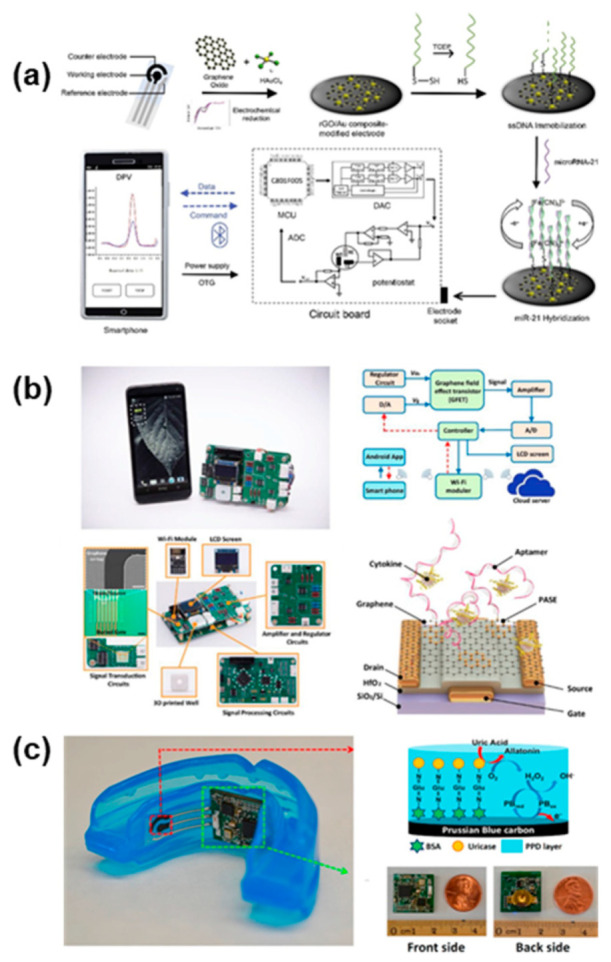
Integrated salivary biosensing systems as POC diagnostic tools for oral cancer biomarkers detection (**a**) Portable biosensing system for the rapid and accurate detection of miRNA in human saliva. Reprinted with permission from ref. [113]. Copyright 2020 Elsevier. (**b**) Handheld fully integrated nano biosensing device for ultrafast cytokine detection in human saliva samples. Reprinted with permission from ref. [101]. Copyright 2019 Elsevier. (**c**) Mouthguard-based biosensing systems with integrated electronics for selective intraoral detection of uric acid. Reprinted with permission from ref. [106]. Copyright 2015 Elsevier.

With the advent of nanotechnology and simultaneous rapid advancements in the field of microelectronics, researchers have been enabled to build complicated advanced devices that would fit in a pocket, or that could even be worn on the body or positioned inside the oral cavity. Electrochemical biosensors possess intrinsic characteristics that make them suitable for scale-down and miniaturization, therefore the vast majority, if not the entirety, of these systems relies on electrochemical transduction. When comparing integrated systems with standalone sensors, such as the one presented in the sections above, it is evident how the former has much more stringent design requirements, as they must fit additional components, such as batteries, microprocessors, and signal conditioning circuits, inside a device with a small form factor. These requirements, often hard to satisfy, have limited the development of these systems, especially wearable devices. Other problems include the degradation of sensitive components, such as bioreceptors (enzymes, antibodies, etc.), that needs to be replaced and frequent calibration of the device, that often cannot be carried out by the final user, thus limiting its applicability in clinical settings. The devices reported in Figure 8 summarize the efforts in the development of wearable and portable devices for the detection of salivary biomarkers associated with oral cancer.

Low et al. [113] recently developed a portable biosensing device (Figure 8a) providing a cost-effective solution for point of care testing of oral cancer onset. The platform has been developed towards the detection of circulating microRNAs, a category of highly stable biomarkers that have been frequently associated with the early onset of malignancies, including oral cancer [152]. Specifically, Low et al. focused on the detection of a salivary microRNA-21 (miR-21), as a first attempt to validate their proof-of-concept. The biosensing systems comprise several elements: the electrochemical sensing strip based on a three-electrodes configuration have been functionalized with reduced graphene oxide (rGO) followed by the immobilization of single strand DNA (ssDNA) as the bioreceptor. The detection of miR-21 hybridization on the electrode surface is performed through DPV thanks to a miniaturized potentiostat that have been included in the customized circuit board, comprising also a microcontroller (MCU), analog-to-digital converter (ADC) and digital-to-analog converter (DAC). An external device, such as a smartphone, supplies the power necessary to operate the biosensing system, and process the data that are transmitted wirelessly through a low-power Bluetooth connection. The compact, handheld device is able to perform rapid detection of miR-21 in human saliva samples, within a linear range of 10^−4^–10^−12^ M.

Another portable biosensing system (Figure 8b) has recently been proposed by Han et al. [101] for the detection of cytokines in human saliva. The group realized a biosensor with integrated readout circuitry and wireless data communication for real-time detection of IL-6. The transduction mechanism relies on a graphene field-effect transistor (GFET) whose working principle and output can be analyzed through I-V curves. The small amount of IL-6, normally present in human saliva only at low concentrations, can be detected as the device operates in the picomolar range. The biosensor has also been tested for selectivity against common interferents, such as human growth hormone (GH) and epidermal growth factor (EGF). Overall, the device showed an acceptable reproducibility and stability, and an exceptionally rapid time to response of only 400 s. The compact form factor and wireless operation makes it a promising device for POC applications in oral cancer diagnostics.

Steering away from portable devices toward wearable biosensing systems, Kim et al. [106] were the first to introduce a fully integrated mouthguard-based biosensing device for the real-time monitoring of uric acid (Figure 8c). The biosensor features a traditional three-electrodes configuration screen-printed onto a plastic (PET) substrate. The flexible sensing pads are interfaced with the integrated circuitry that performs data processing and transmission directly on-board. The tailored enzymatic functionalization allowed for selective sensing of uric acid in presence of common interferents. The group obtained a LOD of 0.35 pM and an exceptionally fast response time of 1 min. The device has been tested both on artificial and real human saliva samples.

**Figure 9:**
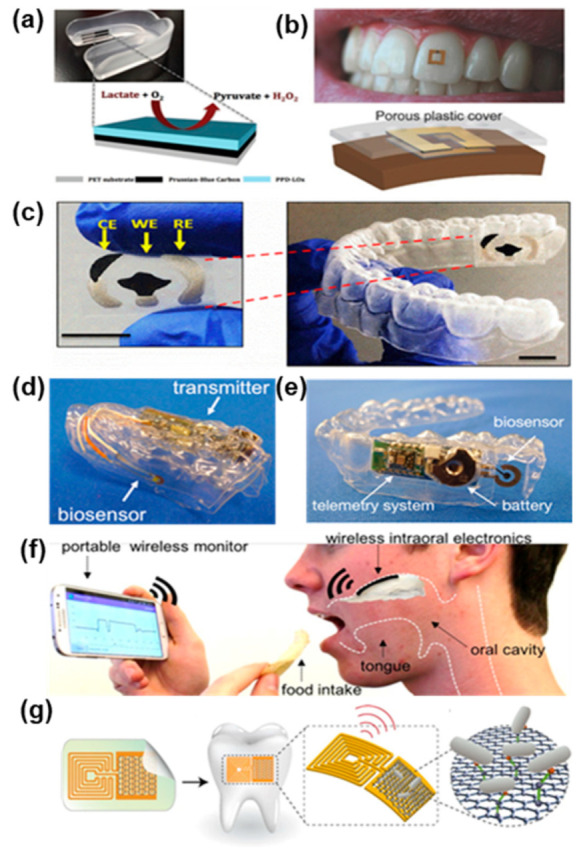
Wearable biosensing platforms for non-invasive salivary analysis (**a**) Lactate biosensor on a mouthguard. Reprinted with permission from ref. [153]. Copyright 2014 Elsevier. (**b**) Tooth mounted RF hydrogel biosensor. Reprinted with permission from ref. [154]. Copyright 2018 Wiley International Limited. (**c**) Mouthguard with screen printed electrodes for N-Carboxymethyl-lysine detection. Reprinted with permission from ref. [155]. Copyright 2019 Elsevier. (**d**,**e**) Glucose biosensing telemetry system. Reprinted with permission from ref. [156]. Copyright 2020 ACS. Reprinted with permission from ref. [157]. Copyright 2016 Elsevier. (**f**) Hybrid flexible bioelectronic platform for sodium monitoring. Reprinted with permission from ref. [160]. Copyright 2018 National Academy of Sciences. (**g**) Graphene-based biosensor for pathogen’s detection. Reprinted with permission from ref. [161]. Copyright 2012 Springer Nature Limited.

The other wearable platforms that constitute the state of the art of intraoral biosensing devices are reported in Figure 9. Kim et al. [153] developed a first printed version of a mouthguard-based biosensor (Figure 9a) before introducing the highly integrated wearable device reported in Figure 8c. Their first attempt featured the same three-electrodes screen-printed biosensing strip, attached onto a mouthguard and tested remotely via wired connection. The development of this early version paved the way for the subsequent implementation of the dedicated circuitry that rapidly followed. A different approach was taken by Tseng et al. [154], who investigated the possibility to attach a sensor directly onto a human tooth. They realized a hydrogel-based resonator (Figure 9b) that could be wirelessly interrogated via RF communication, thus enabling non-invasive food consumption monitoring. Limitations of the systems included the interference of other components, contributing to the swelling of the hydrogel layer, thus reducing the selectivity of the device, and the fragility of the sensor, that is placed in a highly dynamic environment. Figure 9c reports a mouthguard-based biosensor developed by Ciui et al. [155], based on a similar SPCE configuration as the one seen in Figure 9a,c. In this case the screen-printed electrochemical sensor is dedicated to determining the concentration of N-Carboxymethyl-lysine (CML) in saliva, a typically advanced glycation end product, related to oxidative stress and long-term protein damage. This disposable sensor, that can be easily attached onto a mouthguard, demonstrated high flexibility while retaining its integrity. Moreover, it offered an inexpensive, sensitive, and selective tool for the detection of salivary analytes. The group tested the device in vitro and expects to perform real-time in vivo tests in the near future. Arakawa’s work [156,157] on intraoral wearable devices produced two sequential mouthguard-based biosensors, reported in Figure 9d,e. The aim of the devices is to perform continuous and non-invasive intraoral glucose sensing, which the group was able to demonstrate only in their most recent study. The telemetry system features onboard BLE communication for rapid data exchange and time stamping of hyper/hypoglycemia events. The intersection between soft materials and rigid discrete components gave birth to the field of flexible hybrid electronics, that is receiving increasing interest from the scientific community [158,159]. The device reported in Figure 9f, by Lee et al. [160] is an example of flexible hybrid electronics applied to intraoral biosensing. The group developed a retainer with embedded electronics for non-invasive detection of sodium concentration directly in saliva. The ultrathin membrane sensor based on ion-selective electrodes is capable of real-time data transmission to a mobile device thanks to its Bluetooth capability. The group demonstrated both in vitro and in vivo applicability, paving the way for the development of similar devices for the detection of different biomarkers. Wherever time stamping and real-time data recording is not needed, wearable devices can be greatly simplified in terms of their architecture, while contemporarily reducing their form factor. Thin film biosensors, such as the one developed by Mannoor et al. [161] and reported in Figure 9g, exemplifies this concept. A chemi-resistor based on an antibody-immobilized graphene sensing layer works as the transducer, while an RFID inductive coil connected in series to the transducer allows for the wireless interrogation. This smart electronic tattoo can be easily laminated onto complex 3D object or even directly attached onto a tooth, as the authors demonstrate. The low-cost, easy fabrication procedure and high versatility of such systems make them extremely promising for advanced molecular diagnostics at the point of care.

## 5. Conclusions and Future Perspectives

This review provides a comprehensive overview of the most advanced biosensing strategies that have been developed to perform early-stage diagnosis of oral cancer. Risk factors, most common origin sites and diagnostic procedures related to oral cancer have been presented in order to contextualize the urgent need for more sophisticated diagnostic tools. The importance of saliva as a readily available and continuously renovated biofluid has been stressed in order to stimulate further research on salivary biomarkers, whose timely detection could improve patients’ quality of life and their survival rate [16,17,21]. In the present narrative review, we aimed to include all possible molecules and nano biochemical substances, or materials present in saliva and potentially suitable as biomarkers of local/systemic diseases or conditions. However, the origin of these molecules has to be verified before they could be classified as candidate biomarkers. As an example, a recent study has excluded isoprene as a potential metabolic cancer biomarker, thus preventing its applicability to clinical practice [162]. Moreover, many of the studies listed here have been based on a technological developmental approach, involving few subjects and thousands of different variables. In order to translate this basic information into biomarkers for disease prevention and clinical use, new transitional and translational studies are needed as the first step of marker development research. Only through specific studies on technical and biological variability of salivary biomarkers will we be able to control potential systemic and random errors to implement their assessment in clinical research. Well-designed clinical trials on diagnostic, predictive and prognostic performance of saliva biomarkers will complete the path to their application in clinical practice. At time of writing, however, there is no salivary biomarker that can be applied in routine clinical practice. The absence of disease-specific markers that can guarantee values of absolute sensitivity and specificity currently confines the applicability of advanced biosensors targeting biofluids to research settings. The biomarkers that have been reported in the review are thus to be considered being proposed biomarkers that should be targeted in future studies rather than biomarkers having reached maturity for their applicability in clinical practice. The abundance of salivary biomarkers that have been proposed for cancer screening or early diagnosis in the last decade demonstrates a strong interest from the medical community. It is fundamental, however, to validate the findings obtained in pilot studies among larger population groups in order to draw significant correlations between the results. Without substantial validation, it is highly unlikely that clinicians would be willing to introduce advanced biosensing tools based on salivary biomarkers in clinical practice, given the possibility of numerous false positive and false-negative occurrences. Moreover, saliva being a biofluid of highly dynamic composition, confounding factors should be taken into close consideration when carrying out salivary analysis and collection/sampling procedures should be standardized and automated as much as possible to reduce the presence of confounders. In this regard, the use of wearable chemical biosensors could help in tracing the concentration of specific biomarkers throughout prolonged periods of time, while advanced statistical tools can be used within the post-processing steps to minimize the issue. Wearable intraoral bioelectronic platforms and portable POC devices are expected to see an increasing clinical applicability in the near future, as they offer substantial advantages compared to traditional laboratory equipment and procedures. Laboratory techniques require specialized equipment and trained personnel capable of operating these complex systems. This rapidly translates to higher costs associated with each analysis and a longer time-to-response that makes these procedures intricate and not always efficient. Rapid advancements in the field of electronics and nanotechnologies now enable the manufacturing of advanced biosensing systems at a fraction of the cost of complicated laboratory equipment. Given that the concentration of salivary biomarkers is typically lower than that in other biofluids, usually in the ppb-ppt range, extremely sensitivity biosensors are needed to reliably detect the analyte of interest. Limit of detection and sensitivity matches mostly overcome the limits that can be reached with traditional techniques while providing a simpler operation that can be carried out by the user with minimal effort. Moreover, new materials are being researched that can guarantee “green” alternatives to conventional materials within the fabrication process in order to realize more sustainable diagnostic tools. Low-cost, scalable, large-scale manufacturing processes are highly sought to translate research efforts into meaningful practical contributions. The integration of miniaturized wireless communication units within these integrated bioelectronic devices is also a key enabler for the future of smart interconnected systems. In the era of personalized medicine and centralization of sensible data into clouds, it is of utmost importance for the patient to retain property of its own data with enhanced data acquisition and protection systems. Overall, the opportunity to share clinical data with clinicians could enable better predictions based on innovative algorithms based on big data. This would in turn enable a better diagnosis, prognosis, and treatment. We expect intraoral biosensors to play a pivotal role in continuous real-time data acquisition for the detection of potentially malignant biomarkers related to oral cancer. Among the various types of malignancies, oral cancer is best suited to be tackled with wearable sensors placed in the oral cavity due to the direct contact of saliva with premalignant or malignant lesions. Therefore, the aim of this review is that of stimulating research in this direction, while considering the requirements of a translational research that should not only offer interesting new research avenues to pursue inside a laboratory, but also find a sustainable and effective way of developing devices that can have a wide applicability in clinical settings.

## Figures and Tables

**Table 1:** Summary of recent studies of biosensors and bioelectronic systems for early diagnosis of oral cancer (2010-present).

Analyte	Detection	LOD	R/I ^1^ Time	Sample Type	Validation	Ref.
ORAOV-1	SERS ^2^	3.1 fM	60 min	Artificial and real saliva	N/A	[100]
IL-1α	EIS ^3^	6 fg/mL	60 min	Saliva and serum	N/A	[108]
CYFRA 21-1	DPV ^3^	7.2 pg/mL	15 min	Saliva	N/A	[109]
CD63	Fluorescence ^2^	500 particles/μL	30 min	Saliva	NanoFCM	[110]
IL-1β	DPV ^3^	5.2 pg/mL	150 min	Saliva	ELISA	[111]
CYFRA 21-1	SWV ^3^	2.5 ng/mL	N/A	Saliva	ELISA	[112]
miRNA-21	DPV ^3^	1 pM	60 min	Saliva	N/A	[113]
ORAOV-1	PEC ^4^	33 fM	40 min	Saliva	N/A	[114]
HIF-1α	Amperometry ^3^	76 pg/mL	105 min	Saliva	ELISA	[115]
IL-8	DPV ^3^	90 pg/mL	10 min	Saliva	N/A	[116]
S100p mRNA	SERS ^2^	1.1 nM	53 min	Saliva	N/A	[117]
IL-8	DPV ^3^	51.53 pg/mL	10 min	Saliva	N/A	[118]
IL-6	Voltammetry ^3^	12 pM	400 s	Artificial and real saliva	N/A	[101]
IL-1β	EIS ^3^	7.5 fg/mL	45 min	Saliva and serum	N/A	[119]
ORAOV-1	ACV ^3^	12.8 fM	120 min	Artificial saliva	N/A	[102]
CYFRA 21-1	DPV ^3^	0.16 ng/mL	15 min	Saliva	ELISA	[120]
CIP2A	EIS ^3^	0.24 pg/mL	35 min	Saliva	ELISA	[121]
IL-8	EIS ^3^	3.3 fg/mL	45 min	Saliva and serum	ELISA	[122]
IL-1β	EIS ^3^	3 fg/mL	45 min	Saliva and serum	N/A	[123]
CYFRA 21-1	Fluorescence ^2^	0.5 ng/mL	N/A	Saliva	ECL	[124]
IL-8	SFI ^3^	6 fg/mL	45 min	Saliva and serum	ELISA	[125]
TNF-α	Chronoamperometry ^3^	0.001 ng/mL	N/A	Artificial and real saliva	ELISA	[103]
CEA	Colorimetry ^2^	1 ng/mL	1 min	Saliva	N/A	[126]
IL-8	Absorbance ^2^	90 pg/mL	30 min	Saliva	ELISA	[127]
IL-1β	Absorbance ^2^	80 pg/mL	30 min	Saliva	ELISA	[127]
MMP-8	Absorbance ^2^	120 pg/mL	30 min	Saliva	ELISA	[127]
IL-8	DPV ^3^	72.73 pg/mL	9 min	Saliva	N/A	[128]
TNF-α	EIS ^3^	3.7 fg/mL	45 min	Saliva and serum	ELISA	[129]
CYFRA 21-1	DPV ^3^	0.001 ng/mL	5 min	Artificial saliva	ELISA	[104]
IL-1β	Amperometry ^3^	0.38 pg/mL	150 min	Saliva and serum	ELISA	[130]
TNF-α	Amperometry ^3^	0.85 pg/mL	150 min	Saliva and serum	ELISA	[130]
α-amylase	QCM ^5^	1 μg/mL	N/A	Saliva, serum and urine	Phadebas test	[131]
CD59	EIS ^3^	0.84 fg/mL	10 min	Saliva	N/A	[132]
Tryptophan	CC-PSA ^3^	4.9 pM	5 min	Saliva	N/A	[133]
CYFRA 21-1	DPV ^3^	0.01 ng/mL	6 min	Saliva	ELISA	[134]
IL-8	Amperometry ^3^	72.4 pg/mL	5 h	Saliva	ELISA	[135]
IL-8 mRNA	Amperometry ^3^	0.21 nM	5 h	Saliva	ELISA	[135]
CYFRA 21-1	CV ^3^	0.21 ng/mL	15 min	Saliva	ELISA	[136]
CYFRA 21-1	DPV ^3^	0.122 ng/mL	16 min	Saliva	ELISA	[137]
CYFRA 21-1	CV ^3^	0.08 ng/mL	20 min	Artificial and real saliva	ELISA	[105]
Uric Acid	Chronoamperometry ^3^	600 Um	1 min	Artificial and real saliva	N/A	[106]
ORAOV-1	DPV ^3^	0.35 pM	60 min	Saliva	N/A	[94]
IL-8 + TNF-α	FET ^3^	100 fg/mL	N/A	Artificial saliva	ELISA	[107]
IL-6	DPV ^3^	0.39 pg/mL	3 h	Saliva and urine	ELISA	[138]
DNA sequence	Fluorescence ^2^	56 pM	15 min	Saliva and serum	N/A	[139]
hsa-miR-200a	Amperometry ^3^	0.22 aM	100 s	Artificial saliva	N/A	[140]

^1^ R/I = response/incubation. ^2^ Optical. ^3^ Electrochemical. ^4^ Photoelectrochemical. ^5^ Mass-based.

## Data Availability

Data sharing not applicable.
